# Evaluation of the Kaira COVID-19/Flu/RSV Detection Kit for detection of SARS-CoV-2, influenza A/B, and respiratory syncytial virus: A comparative study with the PowerChek SARS-CoV-2, influenza A&B, RSV Multiplex Real-time PCR Kit

**DOI:** 10.1371/journal.pone.0278530

**Published:** 2022-12-14

**Authors:** Tae Yeul Kim, Go Eun Bae, Ji-Youn Kim, Minhee Kang, Ja-Hyun Jang, Hee Jae Huh, Doo Ryeon Chung, Nam Yong Lee

**Affiliations:** 1 Department of Laboratory Medicine and Genetics, Samsung Medical Center, Sungkyunkwan University School of Medicine, Seoul, Korea; 2 Center for Clinical Medicine, Samsung Biomedical Research Institute, Samsung Medical Center, Seoul, Korea; 3 Biomedical Engineering Research Center, Smart Healthcare Research Institute, Samsung Medical Center, Seoul, Korea; 4 Department of Medical Device Management and Research, Samsung Advanced Institute for Health Sciences & Technology, Sungkyunkwan University, Seoul, Korea; 5 Center for Infection Prevention and Control, Samsung Medical Center, Seoul, Korea; 6 Division of Infectious Diseases, Department of Internal Medicine, Samsung Medical Center, Sungkyunkwan University School of Medicine, Seoul, Korea; Waseda University: Waseda Daigaku, JAPAN

## Abstract

**Background:**

Co-circulation of severe acute respiratory syndrome coronavirus 2 (SARS-CoV-2) and other respiratory viruses, such as influenza and respiratory syncytial virus (RSV), can be a severe threat to public health. The accurate detection and differentiation of these viruses are essential for clinical laboratories. Herein, we comparatively evaluated the performance of the Kaira COVID-19/Flu/RSV Detection Kit (Kaira; Optolane, Seongnam, Korea) for detection of SARS-CoV-2, influenza A and B, and RSV in nasopharyngeal swab (NPS) specimens with that of the PowerChek SARS-CoV-2, Influenza A&B, RSV Multiplex Real-time PCR Kit (PowerChek; Kogene Biotech, Seoul, Korea).

**Methods:**

A total of 250 archived NPS specimens collected for routine clinical testing were tested in parallel by the Kaira and PowerChek assays. RNA standards were serially diluted and tested by the Kaira assay to calculate the limit of detection (LOD).

**Results:**

The positive and negative percent agreements between the Kaira and PowerChek assays were as follows: 100% (49/49) and 100% (201/201) for SARS-CoV-2; 100% (50/50) and 99.0% (198/200) for influenza A; 100% (50/50) and 100% (200/200) for influenza B; and 100% (51/51) and 100% (199/199) for RSV, respectively. The LODs of the Kaira assay for SARS-CoV-2, influenza A and B, and RSV were 106.1, 717.1, 287.3, and 442.9 copies/mL, respectively.

**Conclusions:**

The Kaira assay showed comparable performance to the PowerChek assay for detection of SARS-CoV-2, influenza A and B, and RSV in NPS specimens, indicating that the Kaira assay could be a useful diagnostic tool when these viruses are co-circulating.

## Introduction

In December 2019, coronavirus disease 2019 (COVID-19), caused by severe acute respiratory syndrome coronavirus 2 (SARS-CoV-2), emerged in Wuhan, China, and rapidly spread worldwide, achieving pandemic status in March 2020 [1]. As of October 31, 2022, over 627 million people have been infected with SARS-CoV-2 worldwide, resulting in over 6.5 million deaths [2]. To curb the spread of SARS-CoV-2 infection, rapid and accurate laboratory diagnosis is required, and molecular assays are the current gold standard for laboratory diagnosis of SARS-CoV-2 infection [3–5]. More than 200 SARS-CoV-2 molecular assays have been granted emergency use authorization by the US Food and Drug Administration, the majority of which use real-time reverse transcription polymerase chain reaction (rRT-PCR) technology.

During the COVID-19 pandemic, circulation of other respiratory viruses, such as influenza and respiratory syncytial virus (RSV), may pose a tremendous challenge to healthcare systems, as SARS-CoV-2 and these viruses can cause similar symptoms [6–8]. Furthermore, co-infection of SARS-CoV-2 and other respiratory viruses can occur, albeit at a low rate [9, 10]. To address this situation, various molecular assays to simultaneously detect SARS-CoV-2 and other respiratory viruses have been developed and are widely used in clinical settings [11–21].

**Fig 1:**
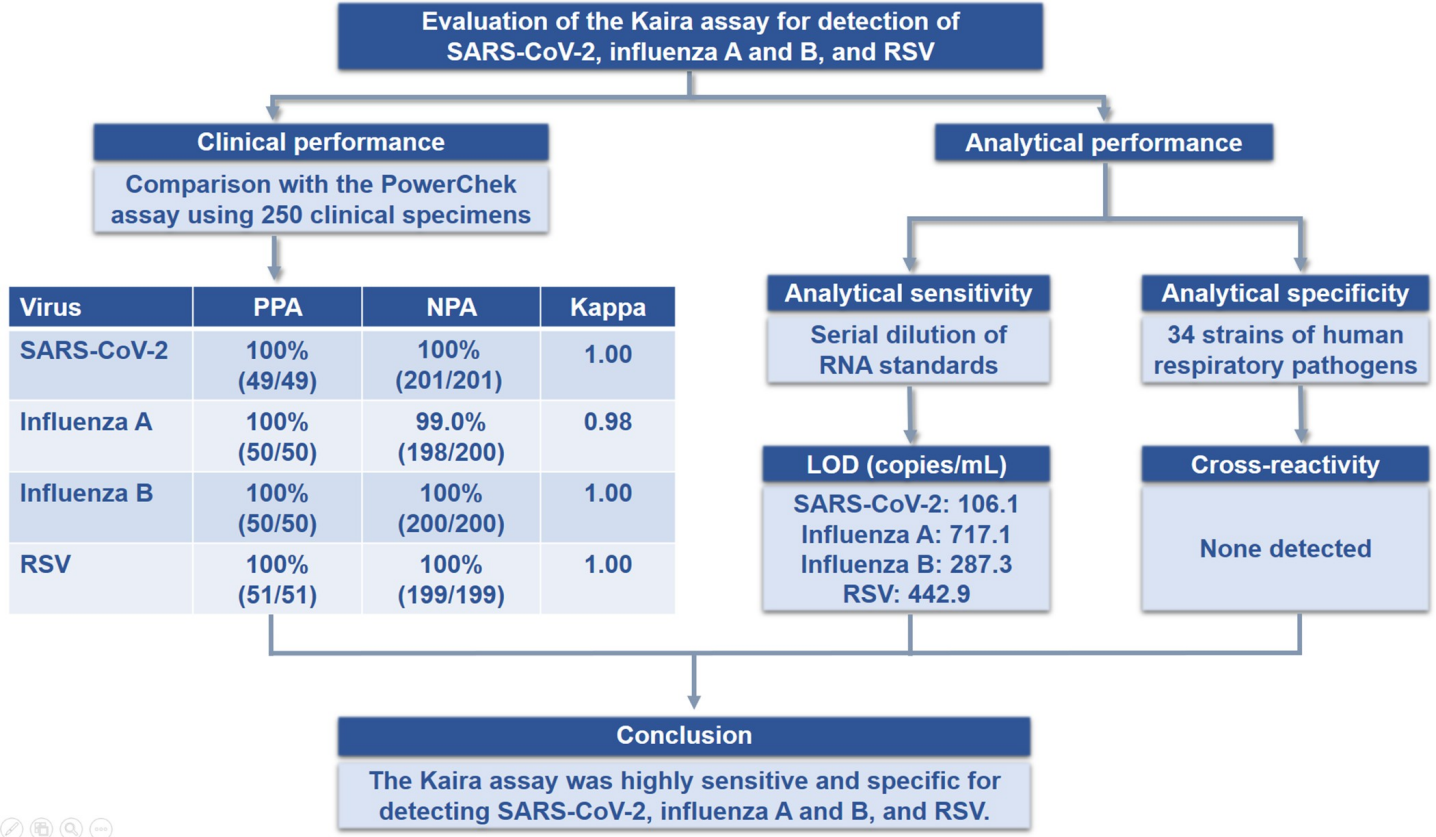
Graphical abstract of the study.

The Kaira COVID-19/Flu/RSV Detection Kit (Kaira; OPTOLANE, Seongnam, Korea) is a novel rRT-PCR assay that can detect SARS-CoV-2, influenza A and B, and RSV in nasopharyngeal swab (NPS) specimens within 80 min. This assay is a single-tube multiplex assay targeting the open reading frame 1ab (ORF1ab) of SARS-CoV-2, the matrix protein 2 gene of influenza A, the nuclear export protein gene of influenza B, and the matrix protein gene of RSV. Herein, we assessed the performance of the Kaira assay compared with the PowerChek SARS-CoV-2, Influenza A&B, RSV Multiplex Real-time PCR Kit (PowerChek; Kogene Biotech, Seoul, Korea). The graphical abstract of this study is shown in Fig 1.

## Material and methods

### Clinical specimens

This study included 250 NPS specimens collected for routine clinical testing at Samsung Medical Center, a 1989-bed tertiary care hospital in Seoul, Korea. Positive specimens were selected based on the cycle threshold (Ct) values obtained from routine clinical testing and covered a wide range of Ct values (Table 1). All specimens were stored frozen at −70°C until retrieved for this study. This study was reviewed and approved by the Institutional Review Board (IRB) of Samsung Medical Center (approval number: 2022-08-010). The need for informed consent was waived by the IRB due to the retrospective study design and use of fully anonymized patient data.

**Table 1:** Distribution of Ct values of positive specimens selected for this study.

	SARS-CoV-2		Influenza virus		RSV
Ct value*	E	ORF1ab	Influenza A	Influenza B	
< 20	13	12	8	15	3
20–25	6	4	20	13	15
25–30	9	11	18	15	18
> 30	22	23	4	7	14
Total no.	50	50	50	50	50

* Ct values were obtained by routine clinical testing using the PowerChek SARS-CoV-2 Real-time PCR Kit (Kogene Biotech) for SARS-CoV-2 and the AdvanSure RV-plus real-time RT-PCR (LG Chem, Seoul, Korea) for influenza and RSV.

E, envelope gene; ORF1ab, open reading frame 1ab.

### Kaira assay

RNA extraction was performed using QIAamp DSP Viral RNA Mini Kit (Qiagen, Hilden, Germany). The Kaira assay was performed according to the manufacturer’s instructions. In brief, 10 μL of template RNA was added to 12.5 μL of rRT-PCR master mix and 2.5 μL of primer/probe mixture, giving a final reaction volume of 25 μL. The rRT-PCR was performed on the CFX96 system (Bio-Rad, Hercules, CA, USA) using the following cycling conditions: 1 cycle at 50°C for 10 min and 1 cycle at 95°C for 10 min, followed by 45 cycles at 95°C for 10 sec and 57°C for 30 sec. For the SARS-CoV-2 target, a Ct value ≤ 42 was considered a positive result, while for the other three targets, a Ct value ≤ 43 was considered a positive result.

### PowerChek assay

The PowerChek assay is a two-tube multiplex rRT-PCR assay and was performed according to the manufacturer’s instructions. In brief, 5 μL of template RNA was added to 10 μL of rRT-PCR master mix and 5 μL of primer/probe mixture, giving a total reaction volume of 20 μL. The rRT-PCR was performed on the CFX96 system using the following cycling conditions: 1 cycle at 50°C for 30 min and 1 cycle at 95°C for 10 min, followed by 40 cycles of 95°C for 15 s and 60°C for 1 min. For all four targets, a Ct value ≤ 38 was considered a positive result. If the results of the Kaira and PowerChek assays were discordant, the BioFire Respiratory Panel 2.1 (RP2.1; bioMérieux, Marcy l’Etoile, France) was conducted.

### Analytical performance

The analytical sensitivity of the Kaira assay was assessed using AMPLIRUN TOTAL SARS-CoV-2/FluA/FluB/RSV CONTROL (SWAB) (Vircell, Granada, Spain). This RNA standard was serially diluted in a pool of negative NPS specimens and extracted as described above. Twenty replicates per dilution level were tested using the Kaira assay.

The analytical specificity of the Kaira assay was evaluated using 34 strains of human respiratory pathogens (Table 2). Bacterial and viral strains were tested in duplicate at concentrations of 1 × 10^6^ and 1 × 10^5^ copies/mL, respectively.

**Table 2:** Analytical specificity evaluation results of the Kaira assay.

Organism	Source (code number)	Result
SARS-CoV-2 B.1.1.7 (Alpha)	Vircell (MBC138-R)	SARS-CoV-2 positive
SARS-CoV-2 B.1.351 (Beta)	Vircell (MBC139-R)	SARS-CoV-2 positive
SARS-CoV-2 P.1 (Gamma)	Vircell (MBC140-R)	SARS-CoV-2 positive
SARS-CoV-2 B.1.617.2 (Delta)	Vircell (MBC141-R)	SARS-CoV-2 positive
SARS-CoV-2 B.1.1.529 (Omicron)	Vircell (MBC143-R)	SARS-CoV-2 positive
SARS-CoV	Vircell (MBC136-R)	Negative
MERS-CoV	Vircell (MBC132)	Negative
Human coronavirus 229E	ATCC (VR-740D)	Negative
Human coronavirus OC43	Vircell (MBC135-R)	Negative
Human coronavirus NL63	Vircell (MBC142-R)	Negative
Human coronavirus HKU1	Clinical isolate	Negative
Influenza A virus H1N1	Vircell (MBC028)	Influenza A positive
Influenza A virus H3N2	Vircell (MBC029)	Influenza A positive
Influenza A virus H5N1	Vircell (MBC052)	Influenza A positive
Influenza B virus	Vircell (MBC030)	Influenza B positive
RSV type A	Vircell (MBC041)	RSV positive
RSV type B	Vircell (MBC083)	RSV positive
Human parainfluenza virus 1	Vircell (MBC037)	Negative
Human parainfluenza virus 2	Vircell (MBC038)	Negative
Human parainfluenza virus 3	Vircell (MBC039)	Negative
Human parainfluenza virus 4	Vircell (MBC050)	Negative
Enterovirus D68	Vircell (MBC125)	Negative
Enterovirus A71	Vircell (MBC019)	Negative
Rhinovirus B14	Vircell (MBC091)	Negative
Human adenovirus 1	Vircell (MBC001)	Negative
Human bocavirus	ATCC (VR-3251SD)	Negative
Human metapneumovirus	Vircell (MBC144-R)	Negative
*Streptococcus pneumoniae*	ATCC (33400D-5)	Negative
*Haemophilus influenzae*	ATCC (51907D-5)	Negative
*Chlamydophila pneumoniae*	ATCC (53592D)	Negative
*Mycoplasma pneumoniae*	ATCC (15531D)	Negative
*Legionella pneumophila*	ATCC (33152D-5)	Negative
*Bordetella pertussis*	ATCC (9797D-5)	Negative
*Bordetella parapertussis*	ATCC (15311D-5)	Negative

### Statistical analysis

Two-by-two tables were used to assess the agreement between the Kaira and PowerChek assays. The positive percent agreement (PPA), negative percent agreement (NPA), Cohen’s kappa values, and two-sided 95% confidence intervals were calculated to evaluate the level of agreement between the two assays. The correlation between Ct values of positive specimens by the two assays was assessed using Pearson correlation coefficient. The limit of detection (LOD) was determined using Probit regression analysis. All statistical analyses were performed using Excel (Microsoft, Redmond, WA, USA) and MedCalc Statistical Software version 19.5 (MedCalc Software Ltd, Ostend, Belgium).

## Results

**Fig 2:**
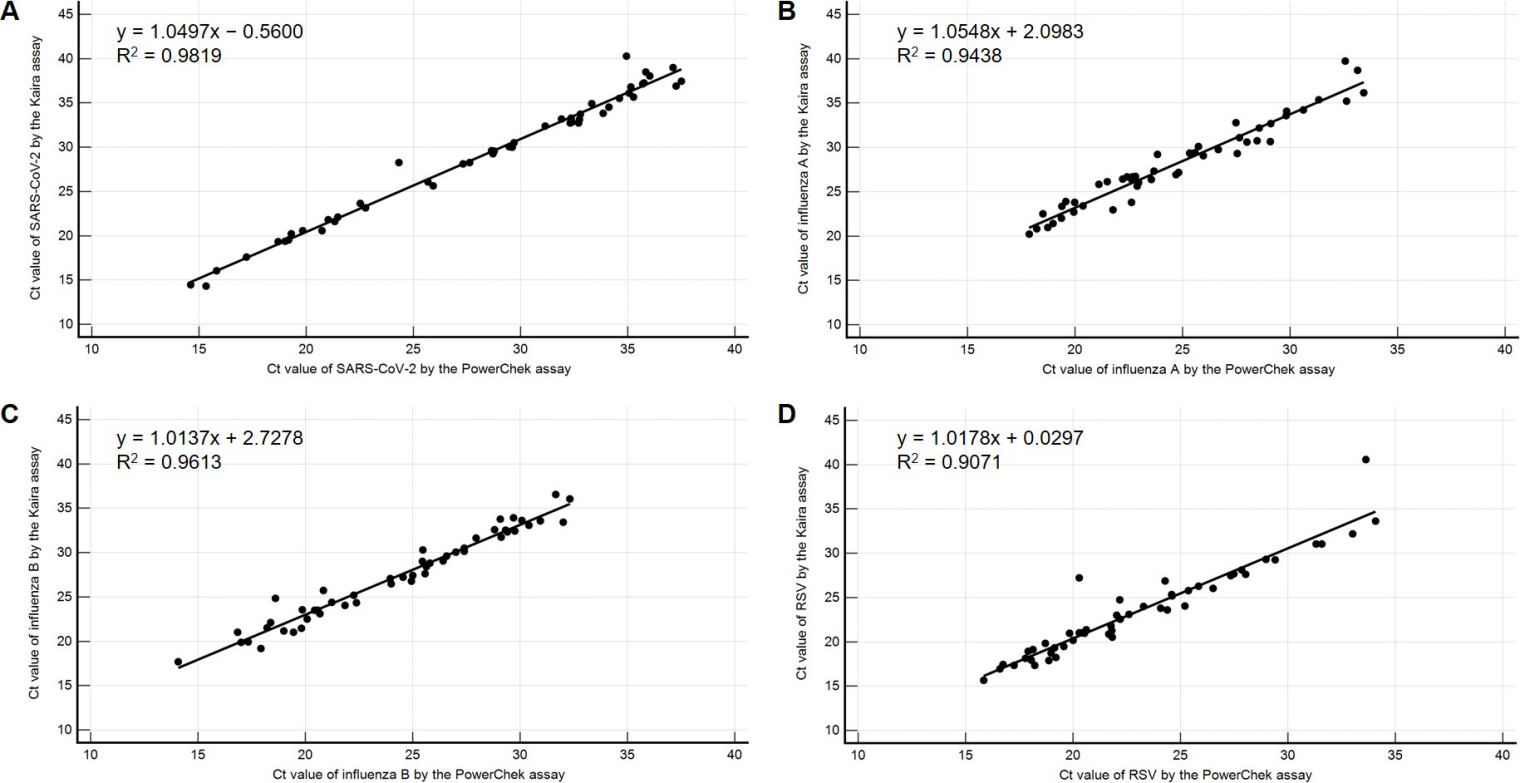
Correlation between Ct values of clinical specimens tested positive by both the Kaira and PowerChek assays. (A) E and ORF1ab Ct values of the PowerChek assay were averaged and plotted against the ORF1ab Ct values of the Kaira assay. (B) Influenza A Ct values of the PowerChek assay were plotted against the influenza A Ct values of the Kaira assay. (C) Influenza B Ct values of the PowerChek assay were plotted against the influenza B Ct values of the Kaira assay. (D) RSV Ct values of the PowerChek assay were plotted against the RSV Ct values of the Kaira assay.

Compared to the PowerChek assay, the PPA and NPA of the Kaira assay for SARS-CoV-2 were 100% (49/49) and 100% (201/201), respectively. The PPA and NPA for influenza A and B were as follows: 100% (50/50) and 99.0% (198/200) for influenza A and 100% (50/50) and 100% (200/200) for influenza B. The PPA and NPA for RSV were 100% (51/51) and 100% (199/199), respectively. Kappa values ranged from 0.98 (influenza A) to 1.00 (SARS-CoV-2, influenza B, and RSV), indicating an almost perfect agreement (Table 3). The Ct values of clinical specimens tested positive by both the Kaira and PowerChek assays were highly correlated, with R^2^ ranging from 0.9071 to 0.9819 (Fig 2). Only two specimens showed discordant results. These specimens tested positive for influenza A and B by the Kaira assay; however, their Ct values for influenza A (42.6 and 42.3) were near the assay cut-off. They tested positive only for influenza B by the PowerChek and RP2.1 assays (Table 4).

**Table 3:** Clinical performance of the Kaira assay in comparison with the PowerChek assay.

Kaira result		PowerChek result		PPA (95% CI)	NPA (95% CI)	Kappa value (95% CI)
		Positive	Negative			
SARS-CoV-2	Positive	49	0	100% (92.7–100%)	100% (98.2–100%)	1.00 (1.00–1.00)
	Negative	0	201			
Influenza A	Positive	50	2	100% (92.9–100%)	99.0% (96.4–99.9%)	0.98 (0.94–1.00)
	Negative	0	198			
Influenza B	Positive	50	0	100% (92.9–100%)	100% (98.2–100%)	1.00 (1.00–1.00)
	Negative	0	200			
RSV	Positive	51	0	100% (93.0–100%)	100% (98.2–100%)	1.00 (1.00–1.00)
	Negative	0	199			

PPA, positive percent agreement; NPA, negative percent agreement; CI, confidence interval.

**Table 4:** Details of two specimens showing discordant results between the Kaira and PowerChek assays.

Specimen no.	Clinical comparison		Discrepancy resolution
	Kaira result (Ct value)	PowerChek result (Ct value)	RP2.1 result
124	Influenza A (42.6)*, Influenza B (22.1)	Influenza B (20.5)	Influenza B
131	Influenza A (42.3)*, Influenza B (27.2)	Influenza B (25.8)	Influenza B

* On repeat testing using the Kaira assay, these specimens showed negative results for influenza A.

The LODs of the Kaira assay for SARS-CoV-2, influenza A and B, and RSV were 106.1, 717.1, 287.3, and 442.9 copies/mL, respectively (Table 5), which were comparable to those of the PowerChek assay determined in our previous study (362.7, 1239.8, 90.2, and 634.4 copies/mL, respectively) [14]. In the analytical specificity test, all intended targets of the Kaira assay (SARS-CoV-2, influenza A and B, and RSV) were detected, and no cross-reactivity with other respiratory pathogens was observed (Table 2).

**Table 5:** Analytical sensitivity evaluation results of the Kaira assay.

Target concentration	SARS-CoV-2			Influenza A			Influenza B			RSV		
	Copies/mL	Replicates	Detected	Copies/mL	Replicates	Detected	Copies/mL	Replicates	Detected	Copies/mL	Replicates	Detected
#1	2500	20	20	3500	20	20	2400	20	20	4000	20	20
#2	1250	20	20	1750	20	20	1200	20	20	2000	20	20
#3	500	20	20	700	20	18	480	20	20	800	20	20
#4	250	20	20	350	20	0	240	20	17	400	20	18
#5	50	20	12	70	20	0	48	20	3	80	20	11
#6	25	20	7	35	20	0	24	20	0	40	20	8
#7	12.5	20	5	17.5	20	0	12	20	1	20	20	2
Probit LOD (copies/mL)	106.1			717.1			287.3			442.9		

## Discussion

In this study, we compared the performance of the Kaira and PowerChek assays for detection of SARS-CoV-2, influenza A and B, and RSV in NPS specimens. We found that the performance of the Kaira assay was comparable to that of the PowerChek assay.

The COVID-19 pandemic has drastically changed the epidemiology of other respiratory viruses. During the 2020–2021 season, other respiratory viruses circulated at historically low levels due to public health measures to curb the spread of SARS-CoV-2. Notably, the circulation of influenza and RSV was virtually absent during this period [22–25]. However, after relaxation of public health measures, an unexpected out-of-season resurgence of influenza and RSV has recently been observed in many parts of the world [26–30]. Given the changes in the epidemiology of influenza and RSV, molecular assays to simultaneously detect these viruses and SARS-CoV-2 are urgently needed and should be performed throughout the year.

Currently, various molecular assays to simultaneously detect SARS-CoV-2 and other respiratory viruses are commercially available, most of which are sample-to-result rRT-PCR assays [11–21]. Sample-to-result assays such as the RP2.1 and Xpert Xpress SARS-CoV-2/Flu/RSV assays are simple to perform and do not require skilled personnel. Furthermore, these assays enable random-access testing, providing test results to physicians in a timely manner; however, they have relatively low throughput and are suited for small-volume clinical laboratories [14, 19]. By contrast, the Kaira and PowerChek assays are designed for high-throughput batch testing (Kaira assay: up to 96 specimens per batch; PowerChek assay: up to 48 specimens per batch) and suited for high-volume clinical laboratories. The performance of the PowerChek assay has recently been evaluated [14]; however, little is known about the performance of the Kaira assay. To the best of our knowledge, this is the first study to evaluate the performance of the Kaira assay.

In this study, the clinical performance of the Kaira assay was comparable to that of the PowerChek assay, with kappa values ranging from 0.98 (influenza A) to 1.00 (SARS-CoV-2, influenza B, and RSV). Only two specimens gave discordant results (Kaira: positive for influenza A and B; PowerChek: positive for influenza B only), which were resolved by the RP2.1 assay (positive for influenza B only). On repeat testing using the Kaira assay, these specimens showed positive results only for influenza B. As the initial Ct values for influenza A were near the assay cut-off and coinfection of influenza A and B viruses is rare [31, 32], the initial positive results for influenza A are highly likely to be false-positive. In addition, the LODs of the Kaira assay were comparable to those of the PowerChek assay, indicating high sensitivity of the Kaira assay in detecting SARS-CoV-2, influenza A and B, and RSV.

An important limitation of the Kaira assay is that it utilizes only one target gene (ORF1ab) for detection of SARS-CoV-2. As mutations in the primer/probe binding sites of the SARS-CoV-2 genome could compromise the rRT-PCR assay’s performance, it is important to use rRT-PCR assays targeting at least two independent regions of the SARS-CoV-2 genome [33–35]. Although the Kaira assay correctly detected all SARS-CoV-2 strains included in this study, clinical laboratories should be aware of this assay’s limitations regarding the use of only one SARS-CoV-2 target.

A major limitation of this study is its retrospective design. A prospective study was not feasible because during the ongoing COVID-19 pandemic, influenza cases, particularly influenza B cases, have rarely been identified in Korea. To obtain a sufficient number of positive specimens, archived NPS specimens previously collected for routine clinical testing were used for this study.

In conclusion, the Kaira assay was found to be highly sensitive and specific for detecting SARS-CoV-2, influenza A and B, and RSV in NPS specimens. During the COVID-19 pandemic, circulation of influenza and RSV may pose a significant challenge to the already overburdened healthcare systems. In this situation, the Kaira assay with a high-throughput capacity (up to 96 specimens per batch) and short turnaround time (80 min) can be useful in clinical settings.

## Supporting information

S1 File Kaira and PowerChek results for each specimen. (XLSX)
